# Deoxycytidine Kinase Augments ATM-Mediated DNA Repair and Contributes to Radiation Resistance

**DOI:** 10.1371/journal.pone.0104125

**Published:** 2014-08-07

**Authors:** Yuri L. Bunimovich, Evan Nair-Gill, Mireille Riedinger, Melissa N. McCracken, Donghui Cheng, Jami McLaughlin, Caius G. Radu, Owen N. Witte

**Affiliations:** 1 Department of Molecular and Medical Pharmacology, University of California Los Angeles, Los Angeles, California, United States of America; 2 Crump Institute for Molecular Imaging, University of California Los Angeles, Los Angeles, California, United States of America; 3 Ahmanson Translational Imaging Division, David Geffen School of Medicine, University of California Los Angeles, Los Angeles, California, United States of America; 4 Howard Hughes Medical Institute, University of California Los Angeles, Los Angeles, California, United States of America; 5 Eli and Edythe Broad Center for Regenerative Medicine and Stem Cell Research, University of California Los Angeles, Los Angeles, California, United States of America; 6 Department of Microbiology, Immunology, and Molecular Genetics, David Geffen School of Medicine, University of California Los Angeles, Los Angeles, California, United States of America; University of Texas Health Science Center at San Antonio, United States of America

## Abstract

Efficient and adequate generation of deoxyribonucleotides is critical to successful DNA repair. We show that ataxia telangiectasia mutated (ATM) integrates the DNA damage response with DNA metabolism by regulating the salvage of deoxyribonucleosides. Specifically, ATM phosphorylates and activates deoxycytidine kinase (dCK) at serine 74 in response to ionizing radiation (IR). Activation of dCK shifts its substrate specificity toward deoxycytidine, increases intracellular dCTP pools post IR, and enhances the rate of DNA repair. Mutation of a single serine 74 residue has profound effects on murine T and B lymphocyte development, suggesting that post-translational regulation of dCK may be important in maintaining genomic stability during hematopoiesis. Using [^18^F]-FAC, a dCK-specific positron emission tomography (PET) probe, we visualized and quantified dCK activation in tumor xenografts after IR, indicating that dCK activation could serve as a biomarker for ATM function and DNA damage response in vivo. In addition, dCK-deficient leukemia cell lines and murine embryonic fibroblasts exhibited increased sensitivity to IR, indicating that pharmacologic inhibition of dCK may be an effective radiosensitization strategy.

## Introduction

Intracellular concentrations of deoxyribonucleotide triphosphates (dNTPs) are tightly regulated to avoid mutagenesis during DNA replication and repair [1]. Mammalian cells synthesize dNTPs by two mechanisms: 1) the *de novo* pathway converts glucose and amino acids to deoxyribonucleotides via ribonucleotide reductase (RNR); 2) the deoxyribonucleoside (dN) salvage pathway generates dNTPs through sequential phosphorylation of recycled deoxyribonucleosides [2]. Deoxycytidine kinase (dCK) is a rate-limiting enzyme in the dN salvage pathway, capable of phosphorylating deoxycytidine (dC), deoxyadenosine (dA) and deoxyguanosine (dG) [3], [4]. Indirectly, dCK can also contribute to dTTP pools via the actions of deoxycytidylate deaminase and thymidylate synthase. Several studies have demonstrated increased dCK activity under various genotoxic conditions, including chemotherapy [5]–[7], ionizing [8]–[10] and UV [11] radiation, and inhibition of several protein kinases [12]–[14]. The potentiation of dCK activity was attributed to post-translational modifications that induced a conformational change of the enzyme [15]–[17]. Phosphorylation of serine 74 (Ser^74^) was shown to be critical in regulating enzyme activity [18]–[20]. dCK can adopt an open state, capable of substrate binding, or a closed, catalytically active, state [21], [22]. Serine to glutamic acid (S74E) substitution mimicking Ser^74^ phosphorylation favors the open state and dramatically reduces phosphorylation of purines (dA and dG) but not pyrimidine dC [22].

Ataxia telangiectasia mutated (ATM) serine/threonine protein kinase is at the center of DNA double-strand break (DSB) repair [23]. ATM is a member of phosphoinositide 3-kinase (PI3K)-related protein kinase family, which also includes ataxia telangiectasia and Rad3-related protein (ATR) and catalytic subunit of DNA-dependent protein kinase (DNA-PKcs) [23]. ATM phosphorylates multiple substrates in the nucleus in response to DNA DSBs [24], and regulates several metabolic pathways which counteract oxidative stress and DNA damage [25]–[29]. In particular, ATM regulates NADPH and ribose-5-phosphate production via the pentose phosphate pathway by promoting phosphorylation of Hsp27, which binds and activates G6PD [25]. ATM also phosphorylates Ser^72^ in the RNR subunit p53R2, which stabilizes the enzyme against degradation and promotes DNA repair [26], [27]. While there is much debate about the purpose of such regulatory mechanisms, it is likely that RNR regulation by ATM is needed to maintain dNTP pools and genomic stability [30].

Evidence from global proteomic analysis identified dCK as a target of ATM based on the phosphorylation of the S^74^Q motif of dCK after ionizing radiation (IR) [31], consistent with recent demonstration of the critical role of dN salvage in DSB repair [32]. While this manuscript was in preparation, Yang et al provided direct evidence for ATM phosphorylation of dCK at Ser^74^ [33]. Phosphorylated dCK was shown to interact with cyclin dependent kinase 1 (Cdk1), thus inhibiting its activity and initiating the G2/M checkpoint. While Yang et al focused on dCK-dependent cell cycle regulation through protein-protein interaction, their work did not address whether ATM modulates the dN salvage pathway through dCK phosphorylation.

Here, we investigate how IR-induced activation of dCK modulates the metabolism of DNA precursors and the affect this has on DNA repair and radiation resistance. In a murine leukemia cell line, we confirm that ATM phosphorylates dCK after IR at Ser^74^. dCK activation shifts its substrate specificity towards dC, resulting in higher rates of intracellular dC sequestration and dCTP production. dCK activation also augments DNA DSB repair, likely through homologous recombination (HR).

Our group has previously developed and characterized positron emission tomography (PET) probes specific for dCK which enable non-invasive measurements of enzyme activity [34], [35]. We utilized one of these probes, [^18^F]-FAC, to visualize dCK activation in subcutaneously implanted tumors following IR. We found that [^18^F]-FAC PET is effective in measuring acute tumor responses to IR, indicating potential clinical utility of this imaging modality in assessing the ATM-mediated DNA damage response in vivo.

Given its emerging importance in DNA DSB repair, we hypothesized that dCK could be a target for new radiosensitizers. A murine leukemia cell line lacking dCK was more radiosensitive than isogenic cells with the restored levels of dCK. Mouse embryonic fibroblasts (MEFs) derived from dCK KO mice also demonstrated enhanced sensitivity to IR. Inhibitors of dCK may prove useful in sensitizing tumors to radiotherapy.

Finally, we hypothesized that endogenous cellular genotoxic stress could also trigger post-translational activation of dCK at Ser^74^. dCK knockout (KO) mice exhibit specific partial blocks in the early stages of T and B lymphocyte development [36]. Furthermore, complete dCK inactivation in mice induces endogenous DNA damage in lymphoid and erythroid lineages [37]. We attempted to understand whether post-translational modification of dCK at Ser^74^ plays a role in the development of B and T lymphocytes. To that end, we performed bone marrow transplantations (BMT) using stem cells from dCK KO bone marrow expressing wild type (WT) dCK or Ser^74^ mutated isoform. Our results demonstrate that dCK Ser^74^ is critical for normal B and T cell development in murine bone marrow transplant model, suggesting that the regulation of dCK at that residue may occur during hematopoiesis in response to physiologic stress.

## Materials and Methods

### Ethics Statement

All animal studies were carried out according to the guidelines of the Department of Laboratory Animal Medicine (DLAM) and the Animal Research Committee at UCLA (Protocol number: ARC 2005-072). All surgery was performed under appropriate anesthesia, and all efforts were made to minimize suffering.

### Cell Lines and Reagents

All reagents were purchased from Sigma-Aldrich unless stated otherwise. The murine leukemia line (L1210-10K) was a previous gift from Dr. Charles Dumontet (Universite Claude Bernard Lyon I, Lyon, France) [38]. The amphotrophic retrovirus packaging cell line 293T was used for the production of murine stem cell virus-based (MSCV) retroviruses [39] containing YFP or dsRed and human or mouse dCK (WT or Ser^74^ mutant). Cell sorting based on color marker using flow cytometry ensured a pure population of dCK-expressing cells that were matched to the same fluorescence intensity. All L1210-10K derived cell lines were cultured in RPMI medium 1640, supplemented with 5% FBS and 2 mM L-glutamine, at 5% CO_2_ and 37 °C. ATR-defective (ATR-Seckel) cell line DK0064 (ATR^A2101G^), wild-type human lymphoblastoid cell line CHOC6, and A-T cell lines (AT224LA^G170A/1402delAA^ and AT255LA^IVS42+2delT/A9171T^) were a gift from Dr. Richard Gatti (University of California, Los Angeles) [40].

### Cloning and Mutagenesis of Deoxycytidine kinase

Total RNA was isolated from mouse thymus and cDNA was generated using RT-PCR (superscript III) with oligo dT priming. Human dCK triple mutant (A100V, R104M, D133A) was codon optimized and synthesized by DNA 2.0, Inc. Triple mutant was mutated back to wild type (5′-GGAGTTTTACTTTTCAAACCTACGCTTGTCTGTCACGAATCAGAGCTCAACTGGCAAGCCTC-3′ and 5′-CTTTGAACGGTCTGTGTATAGTGACAGATACATTTTCGCTTCTAACCT-3′) using a multi-site mutagenesis kit (Agilent). Serine 74 was mutated to glutamic acid (S74E, human 5′-GAGTTCGAAGAGCTGACAATGGAACAGAAGAATGGAGGTAACGTC-3′, mouse 5′-GGAATTTGAGGAATTGACAACGGAGCAGAAGAGCGGTGGAAATGTTC-3′) or to alanine (S74A, human 5′- GTTCGAAGAGCTGACAATGGCTCAGAAGAATGGAGGTAAC-3′, mouse 5′- TGAGGAATTGACAACGGCTCAGAAGAGCGGTGG-3′). dCK was cut out with EcoRI and XhoI and ligated into MSCV-IRES-YFP, MSCV-IRES-DsRed and MSCV-6His-IRES-YFP.

### Generation of Deoxycytidine Kinase Mouse Monoclonal Antibodies

Human dCK (6-His-tagged) was produced in bacteria, purified by Ni-NTA chromatography and used as an immunogen. Balb/c 6–8 week-old female mice were immunized by an intraperitoneal injection of 200 µg of 6-His-dCK in RIBI adjuvant (Sigma), followed by 4 monthly boosts of 100 µg of immunogen. Antibody titers were determined in the serum by ELISA. Spleens of the highest titer mice were excised and dissociated. Isolated splenocytes were fused to sp2/0 myeloma cells at a ratio of 5∶1 splenocytes/myeloma using PEG1500 (Roche). Twenty percent of fusion was plated in HAT medium in 10x flat bottom 96-well plates at 200 µl/well. Fusion was cultured until 25–50% coverage of wells was achieved. Positive wells, determined by ELISA of the supernatant, were re-plated in 24-well plates in HT medium. Supernatants were screened by repeat ELISA and Western blot at 1∶10 dilution in 5% milk/PBS-T. Positive wells were sub-cloned by limiting dilution, and the sub-clones were tested by ELISA and Western blot. Clones with the highest affinities were 3B1, 3E10, 6B9, 9D4, 10A1 and 10H10. Variable region of each monoclonal line was cloned and sequenced to identify individual clones. Clones 3E10, 6B9 and 9D4 have an identical sequence, while 3B1, 10A1 and 10H10 are unique clones. Preparative amounts of antibody were produced in CELLine-1000 flasks (Integra Bioscience) and purified by Prosep-G affinity chromatography (Millipore). Clone 9D4 is commercially available from Millipore.

### Antibodies and Western Blotting

Protein content was measured with BCA assay (Pierce), 5–30 µg was subjected to SDS/PAGE on 4–20% acrylamide gel (Thermo Scientific) followed by transfer to nitrocellulose membrane. When detecting ATM, 7.5% Tris-HCl gel (Bio-Rad) was used, and protein was transferred to PVDF membrane (Millipore) overnight at 30 V. Membranes were blocked with 5% milk in TBS-T (Tween 0.1% wt/vol), and probed with the following antibodies: p53 pSer^15^ (1∶1000, Cell Signaling), p53 (1∶1000, Cell Signaling), ATM pSer^1981^ (1∶1000, Cell Signaling), α-tubulin (1∶2000, Santa Cruz), ERK2 (1∶2000, Santa Cruz), dCK (Clone 9D4, 1∶1000, Millipore). Polyclonal rabbit antibody against dCK pSer^74^ was a gift from Dr. Francoise Bontemps (Universite Catholique de Louvain, Brussels, Belgium). ECL substrate (Millipore) was used for detection and development on GE/Amersham film. For separating nuclear and cytoplasmic lysates, NE-PER Extraction Reagents kit was used (Thermo Scientific).

### *In Vitro* Uptake and Kinase Assay

*In vitro* uptake and kinase assay protocols were adapted from a previous report [41]. The following tritium-labeled compounds were purchased from Moravek Biochemicals: [5-^3^H(N)]-2′-deoxycytidine (22 Ci/mmol), [2,8-^3^H]-deoxyadenosine (8 Ci/mmol), [8-^3^H]-2′-deoxyguanosine (6.6 Ci/mmol). Cells were irradiated using Cs-137 source at a rate of 7.16 Gy/min to a total dose of 3 Gy. Irradiated and untreated cells were placed in wells of a 96-well 0.22-µm MultiScreen filter bottom plates (Millipore) in triplicate, at 1×10^5^ cells/well in 100 µl of 5% RPMI, and 1 µCi of [^3^H]dC was added to each well. Cells were incubated at 37°C, 5% CO_2_ for 1 hour, and washed five times with 200 µl of 5% RPMI using the Multiscreen vacuum manifold (Millipore). Plates were dried, wells were punched out into Wheaton scintillation Omni-Vials (Fisher), and Bio-Safe NA scintillation fluid was added (RPI Research Products). Radioactivity was determined with Beckman scintillation counter (LS 6500). Kinase assays were performed on either total cell lysates or Ni-NTA agarose (Qiagen) bound 6-His-dCK. Lysis buffer was prepared by adding fresh complete protease inhibitors (Roche) and Phosphatase Inhibitor Cocktails 2/3 (1∶100, Sigma) to 50 mM Tris-HCl, 20% glycerol, 0.5% Nonidet P40, pH 7.6. Lysates were cleared by spinning at 16000×g, 4 °C for 20 min. One µl of 1 µg/µl total cell protein was added in triplicate to wells of a 96-well Microtest U-bottom plate (Becton Dickinson) containing 6 µl H2O and 2 µl of 5× kinase buffer (250 mM Tris-HCl, 5 mM ATP, 25 mM MgCl_2_, 10 mM DTT, 50 mM NaF, 5 mM thymidine, pH 7.6). One µCi of an appropriate tritium labeled deoxynucleoside was added to each well and plates were incubated at 37°C for 20 minutes. Reactions were quenched with 40 µl ice cold water, heated at 95°C for 2 minutes, and contents of each well were blotted on Whatman DE-81 filter discs (GE Healthcare). Dried disks were washed three times with 4 mM ammonium formate and twice with 95% ethanol. Radioactivity from dried disks was determined as above. In some cases, prior to irradiation cells were pretreated for 1 hour with ATM inhibitor Ku55933 (Tocris) or DNA-PK inhibitor Nu7441 (Tocris) diluted in DMSO.

### His-Tagged dCK Purification

Cell lysates (650 µg total protein) were diluted 1∶2 with Ni-NTA compatible buffer (40 mM NaH_2_PO_4_, 20% glycerol, 130 mM NaCl, 0.5% NP40, 10 mM imidazole) to which Phosphatase Inhibitor Cocktails 2/3 (1∶100, Sigma) were added. Eighty µl of 50% Ni-NTA agarose beads (Qiagen) were washed with Ni-NTA buffer, added to the lysate and rotated overnight at 4°C. Beads were washed 4 times with Ni-NTA buffer. Equivalent of 50 µg of initial lysate was kept for Western blotting, and the remaining 600 µg equivalent was divided into 150 µg aliquots. dCK kinase assay was performed on each aliquot by adding 10 µl H_2_O_2_, 3 µl 5× kinase buffer and 1 µCi of tritium-labeled deoxyribonucleoside, as described in the *In Vitro Uptake and Kinase Assay* section.

### DNA Repair Assays

L1210-10K cells ± dCK (WT, S74A, S74E) were irradiated using a Cs-137 source at a rate of 7.16 Gy/min to a total dose of 3 Gy and kept in 15 ml Falcon tubes (BD Biosciences) at 37°C, 5% CO_2_. At different time points after irradiation 2×10^5^ cells were taken out and either cytospun onto a glass slide or fixed in 70% ice-cold ethanol overnight. Cytospun cells were fixed, and stained overnight at 4°C with anti-γH2A.X pSer^139^ -Alexa647 mAb (1∶50, Cell Signaling). Ethanol-fixed cells were washed 3 times in PBS and stained overnight at 4°C with anti-γH2A.X pSer^139^ -Alexa647 mAb (1∶100) in FACS buffer (PBS +3% FBS, 0.09% NaN_3_) with 0.1% Saponin. Cells were washed and resuspended in FACS buffer, and analyzed on FACSCanto flow cytometer (BD Biosciences). DNA recombination efficiency was measured using a transient transfection assay described previous [42], [43]. Vector constructs pCMS-end and pCMS-hom to measure non-homologous end-joining (NHEJ) and homologous recombination (HR), respectively, were a gift from Dr. Robert Schiestl (University of California, Los Angeles). Digestion of 500 µg plasmid DNA was carried out with XhoI/BamHI (pCMS-end) or XhoI (pCMS-hom) in 800 µl volume at 37°C for 3–4 hours. Digestion was extracted with 800 µl of phenol:chloroform:isoamylalcohol (25∶24:1) and precipitated with sodium acetate (pH 5.2, 300 mM final) and 2 ml ethanol. Digested plasmids were pelleted at 16000×g, 4°C, washed once with 70% ethanol, dried briefly at 37°C, and re-suspended overnight in 120 µl of TE buffer at 4°C. L1210-10K cells ± dCK/dsRed were irradiated with 3 Gy, and incubated at 37°C and 5% CO_2_ for 1 hour. Nucleofection was performed after combining 18 µg of digested plasmid with 5×10^5^ cells in 100 µl Cell Line Nucleofector Solution V (Lonza), using program S018 on Nucleofector II (Amaxa). Immediately after nucleofection, cells were placed in pre-warmed 1.5 ml 5% RPMI media and incubated at 37°C, 5% CO_2_ for 24 hours. Cells were analyzed on FACSCanto flow cytometer under YFP-GFP-RFP configuration.

### Intracellular dNTP Pool Measurements

L1210-10K cells ± dCK (WT, S74A, S74E) were irradiated using a Cs-137 source at a rate of 7.16 Gy/min to a total dose of 3 Gy and kept in 15 ml Falcon tubes (BD Biosciences) at 37°C, 5% CO_2_. Each hour after irradiation, 1×10^6^ cells were taken out, washed once with PBS, pelleted and re-suspended in 500 µl of ice-cold 60% methanol. Cells were vortexed for 1 min and stored at −20°C overnight. Next day lysates were exposed to 95°C for 3 minutes, and pelleted at 17,000×g for 15 minutes. Supernatants were evaporated for 4 hours in Speed VacPlus SC110A (Savant), and pellets were re-suspended in 100 µl of nuclease-free H_2_O. Previously described protocol was used to measure dNTP pools [44], with the following modifications. Reactions were carried out simultaneously in triplicate in 96-well U-bottom plates (Becton Dickinson) using 5 µl of lysate in 25 µl total volume per well. After incubation for 2 hours at 37°C, 20 µl from each well were transferred to 96-well DE81 Unifilter plates (Whatman). Plates were washed 4 times with Na_2_HPO_4_, once with diH_2_O and once with 95% ethanol using Multiscreen vacuum manifold (Millipore). Washed plates were dried, and 100 µl of scintillation fluid was added to each well. Radioactivity was measured with a BetaMax plate reader (PerkinElmer).

### Bone Marrow Transplantation

Eight to twelve-week-old female dCK KO mice injected intraperitoneally with 150 mg/kg (200 µl) of 5-Fluorouracil (APP Pharmaceuticals) and four days later bone marrow was harvested. Bone marrow was cultured, stimulated with cytokines and infected with a retrovirus as previously described [45]. Ecotropic murine stem cell virus-based retrovirus containing either mouse WT or Ser^74^ mutant dCK was generated in 293T cells with titers of 1–2×10^7^/ml. B6.SJL 8–10 week-old female mice were irradiated using Co-60 source at a rate of 0.177 Gy/min to a total lethal dose of 950 rads, and 3 hours later 200 µl of HBSS containing 3–5×10^5^ infected bone marrow cells were injected into their tail veins. Bone marrow was allowed to engraft for 5–8 weeks.

### Thymus and Bone Marrow Analysis

Thymi, spleens and long bones were harvested from B6.SJL donors after 5–8 weeks of transplantation. Thymi and spleens were dissociated into single cells in 5% RPMI using frosted glass slides, and filtered through 40 µm sterile filters. Bone marrow single cell suspensions were obtained by flushing femur and tibia with 5% RPMI and filtering through 40 µm filters. Cells were washed in FACS buffer (PBS +3% FBS, 0.09% NaN_3_) and counted. Dead cells were excluded by staining with DAPI (50 ng/ml). Antibodies were purchased from eBioscience, Inc. unless stated otherwise. Cell sorting and analysis was performed on FACSAria II flow cytometer (BD Biosciences). Single cell suspensions from spleens were stained with CD45.2-Alexa780 (donor marker, Clone 104) and CD45.1-PE-Cy5 (recipient marker, Clone A20) antibodies, and CD45.2^+^YFP^+^/CD45.2^+^YFP^−^ cells were sorted for Western blotting and dCK kinase assay. CD45.1^+^ cells from a spleen of untreated B6.SJL mouse were sorted as control of dCK expression for Western blot. Thymocyte phenotyping was performed with the following antibodies: CD45.2-Alexa780 (Clone 104), CD45.1-PE-Cy5 (Clone A20), CD4-PE (Clone RM4-5), CD8-PE-Cy7 (Clone 53-6.7), CD25-Alexa700 (Clone PC61.5), CD44-PerCP-Cy5.5 (Clone IM7). Phenotyping of B cell development in the bone marrow was performed with the following antibodies: CD45.2-Alexa780 (Clone 104), CD45.1-PE-Cy5 (Clone A20), B220-PE-Cy7 (Clone RA3-6B2), IgM-biotin (Clone 11/41)+SA-eFluor710, CD43-PE (Clone S7, BDBiosciences), CD19-PerCP-Cy5.5 (Clone 1D3). Approximately 1–6×10^6^ stained live thymocytes and bone marrow cells were pelleted, resuspended in 100 µl of Cytofix/Cytoperm buffer (BD Biosciences) and incubated on ice for 20 minutes. Cells were washed with 1 ml of 1× Perm/Wash buffer (BD Biosciences), re-suspended in 100 µl of Cytoperm Plus buffer (BD Biosciences) and incubated on ice for 10 minutes. Cells were washed, pelleted and stained with γH2A.X pS^139^-Alexa647 antibody (1∶10, Clone 20E3, Cell Signaling) in 50 µl of Perm/Wash buffer overnight at 4°C. Cells were again stained with DAPI (4 µg/ml) in FACS buffer and analyzed.

### *In Vivo* Treatment Model

L1210-10K ± dCK (WT, S74A, S74E) cells were resuspended in fresh RPMI media with no additives at a density of 4×10^7^ cells/ml. Matrigel matrix (BD Biosciences) was added at a 1∶1 volume ratio. NSG and CB17 SCID mice were anesthetized with an intraperitoneal injection of 1.2 mg ketamine/0.38 mg xylazine (Phoenix Pharmaceutical), and either the right or both front legs were injected s.c. with 2×10^6^ cells in 100 µl volume. Tumors were allowed to develop for 10–15 days. Prior to irradiation, mice were anesthetized with an IP injection of ketamine/xylazine, placed on a platform and shielded with a cerrobend jig, except for a right front leg harboring a tumor. Exposed tumors were irradiated using a Gulmay RS320 x-ray unit filtered with 1.5 mm Cu and 3 mm Al (Gulmay Medical) at a rate of 1.17 Gy/min, 300 kV and 10 mA. The x-rays were administered vertically with a focus-to-surface distance of 42.3 cm.

### MicroPET/microCT Imaging and Image Analysis

All mouse imaging was conducted at UCLA Crump Institute for Molecular Imaging - Small Animal Imaging Center as previously described [46]. [^18^F]-FDG was synthesized at the UCLA Ahmanson TranslationalImaging Division biomedical cyclotron facility as previously described [47]. [^18^F]-FAC was synthesized at the Crump Preclinical Technology Center cyclotron facility as previously described [34]. Mice were kept warm under gas anesthesia (2% isoflorane). Approximately 200 µCi of either probe was injected into a tail vein 1 hour prior to the initiation of imaging. To look at the uptake of both probes after irradiation, probes were injected 1 hour after IR. Images were acquired with Siemens Preclinical Solutions microPET Focus 220 and MicroCAT II CT. Image reconstruction and registration was performed as previously described [34]. AMIDE software was used for image analysis [48]. Three dimensional regions of interest (ROI) were drawn around tumors to quantify their volumes and accumulation of the probe as a mean percentage of injected radioactivity dose per weight (%ID/g).

### Radiosensitivity Assays

Clonogenic survival assay was performed on MEFs from dCK KO and litter matched control dCK WT mice. Cells were plated at 300,000 cells/well at 37°C, 8% CO_2_ in six well plates after Cs-137 irradiation. *In vivo* radiosensitivity assay was performed by implanting L1210-10K ± dCK cells into right front legs of 10-week-old CB17 SCID female mice and allowing the tumors to develop for 10 days. Tumors were measured daily with calipers, and volumes were estimated from the formula (L×W^2^)/2. Selective tumor irradiation with 3 Gy was performed twice daily (7 hours apart) for six days as described in *In Vivo Treatment Model* section. Tumors were then allowed to re-grow for 10 days. Imaging with [^18^F]-FDG was performed as described in *MicroPET/MicroCT Imaging and Image Analysis* section.

### Statistical Analyses

Data are presented as means ± SEM. Group comparisons were performed with one-sample t test function in column statistics of Prism 5 software (GraphPad Software). All P values are two-tailed, and P<0.05 was considered to be statistically significant. Graphs were generated with Prism 5 software.

## Results

### dCK activity increases after IR in an ATM-dependent manner and is correlated with Ser^74^ phosphorylation

**Figure 1:**
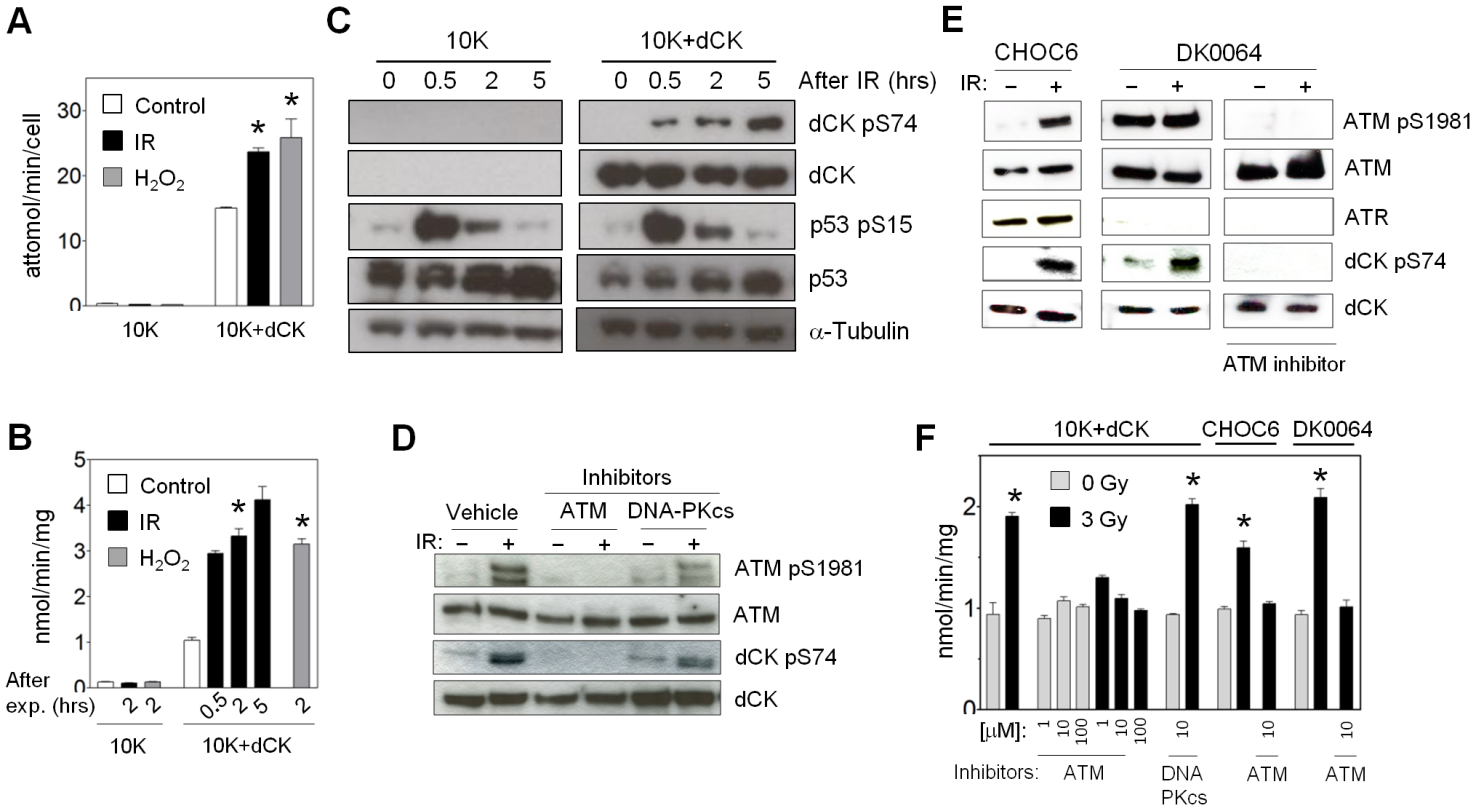
dCK is activated in an ATM-dependent manner after IR. (A) *In vitro* cell uptake assay of [^3^H]-dC 2 hours after exposure of L1210-10K ± dCK (WT) to 3 Gy (*, P = 0.0002; N = 3) or 100 µM H_2_0_2_ (*, P = 0.0197; N = 3). (B) *In vitro* dCK kinase assay using L1210-10K ± dCK (WT) cell lysates, [^3^H]-dC as substrate and performed at indicated times after exposure to 3 Gy (*, P = 0.0002; N = 3) or 100 µM H_2_0_2_ (*, P<0.0001; N = 3). (C) Western blot of L1210-10K ± dCK (WT) cell lysates obtained at indicated times after 3 Gy irradiation. (D) Western blot of lysates from 10K+dCK (WT) cells treated for 1 hour with either DMSO vehicle, 10 µM ATM inhibitor (Ku55933) or 10 µM DNA-PKcs inhibitor (Nu7441) and irradiated with 3 Gy. (E) Western blot of lysates from CHOC6 (WT LCL) and DK0064 (ATR-defective) cell lines before IR or 2 hours after 3 Gy exposure. Lysates from DK0064 cells pretreated with 10 Gy exposure. Lysates from DK0064 cells pretreated with 10 µM ATM inhibitor Ku55933 are shown in the right panel. (F) *In vitro* dCK kinase assay using 10K+dCK (WT), CHOC6 and DK0064 cell lysates, [^3^H]-dC as substrate and performed where indicated after 1 hour pretreatment with inhibitors of ATM and DNA hour pretreatment with inhibitors of ATM and DNA-PKcs, and 2 hours after exposure to 3 Gy (*, 10K+dCK: P<0.0001; CHOC6: P = 0.001; DK0064: P = 0.0003; N = 3).

A large body of evidence has documented the activation of dCK after cell exposure to various genotoxic stresses [5]–[11]. IR is a quantifiable and reproducible exogenous source for generating DNA DSBs, and it is used to treat multiple types of cancer. We aimed to understand the mechanism of dCK regulation resulting from IR. To that end we chose to work with the gemcitabine-resistant L1210-10K cell line derived from a murine leukemia [38]. L1210-10K (10K) cells have a sequencing-confirmed mutation in the dCK gene which leads to a complete absence of the kinase [38]. By reintroducing dCK into 10K cells (10K+dCK) through retroviral transduction, we restored the dCK-dependent branch of the deoxyribonucleoside salvage pathway (Figure 1A). Two hours after irradiation of 10K+dCK cells with 3 Gy the rate of uptake of tritium-labeled dC (^3^H-dC) from the media increased 1.5-fold (Figure 1A). Thirty minutes after irradiation the activity of dCK increased 3 to 4-fold, and remained elevated for 5 hours (Figure 1B). Ionizing radiation leads to oxidative stress through the formation of free radicals [49]. We treated 10K+dCK cells with hydrogen peroxide and observed a 2-fold increase in ^3^H-dC uptake and 3-fold increase in dCK kinase activity (Figure 1A and B). Thus, both IR and oxidative stress induce the activation of dCK and stimulate the salvage of exogenous dC.

Protein from 10K+dCK cells was isolated at time-points after irradiation with 3 Gy. Increased dCK activity correlated with the phosphorylation of the enzyme at Ser^74^, while the total amount of dCK remained unchanged (Figure 1C). Activation of the IR-induced DNA damage response was confirmed by measuring phosphorylation of p53 at Ser^15^ (Figure 1C), a target of DNA-PKcs, ATM and ATR [50]. Phosphorylation of p53 peaked at 30 minutes after IR and returned to baseline by 5 hours (Figure 1C). Our results confirm previous studies showing enhanced dCK activity after IR and demonstrate a direct correlation between IR-induced increase in enzyme activity and phosphorylation at Ser^74^.

ATM is a serine/threonine protein kinase that is a dominant regulator of the cellular response to DNA damage and is critical to the repair of DSBs resulting from IR [23]. Using a large-scale proteomics screen, Matsuoka, et al implicated dCK as a substrate of ATM after IR-induced DNA damage response [31]. While this manuscript was in preparation, Yang et al demonstrated a direct phosphorylation of dCK by ATM at Ser^74^ in response to IR [33]. We also tested whether chemical inhibition or mutation of ATM would block dCK activation by IR. Specific inhibition of ATM in 10K+dCK cells by Ku55933 [51] prevented IR-induced ATM autophosphorylation of Ser^1981^ (Figure 1D). Significantly, pharmacologic inhibition of ATM abrogated IR-induced dCK phosphorylation at Ser^74^ (Figure 1D). ATM inhibition also blocked the IR-induced increase in dCK activity in a dose-dependent manner (Figure 1F).

DNA-PKcs shares functional redundancy and overlapping substrates with ATM [23]. We tested whether dCK was activated after IR in the context of DNA-PKcs inhibition. Treatment of 10K+dCK cells with DNA-PK specific inhibitor Nu7441 [52] demonstrated partial reduction in phosphorylation of ATM at Ser^1981^ and dCK at Ser^74^ (Figure 1D), while the dCK activation after IR was completely preserved (Figure 1F).

ATM and ATR also share substrate specificity, but while ATM senses double-strand breaks, ATR senses single-strand DNA present during DSB processing or at stalled replication forks [53]. We tested IR-induced dCK activation in an ATR-defective (DK0064) human lymphoblastoid cell line (LCL) and compared it to the wild-type (WT) LCL CHOC6. Significant reduction in the level of ATR protein in ATR-defective human LCL compared to WT LCL was confirmed by western blot (Figure 1E). IR-induced phosphorylation of dCK at Ser^74^ was observed in the ATR-defective and WT LCLs (Figure 1E), corresponding to a 2.25 fold and 1.6 fold increase in dCK activity, respectively (Figure 1F). Selective chemical inhibition of ATM with Ku55933 in these LCLs completely abrogated IR-induced ATM autophosphorylation of Ser^1981^, dCK phosphorylation at Ser^74^ (Figure 1E) and dCK activation (Figure 1F). Interestingly, increased endogenous ATM phosphorylated at Ser^1981^ was observed in ATR-Seckel cells compared to WT LCL (Figure 1E).

We have utilized two human A-T cell lines (AT224LA and AT255LA) to confirm the dependence of IR-induced dCK activation on ATM. These cell lines exhibit either a complete absence or a significant reduction of detectable ATM protein, respectively (Figure S1A). Two hours after irradiation the uptake of ^3^H-dC was unchanged in A-T cells (Figure S1B), and the activation of dCK was significantly reduced compared to WT and ATR-defective LCLs (Figure S1C). In sum, these results utilizing a murine leukemia cell line model and human A-T LCLs confirm that the activation of dCK in response to IR is ATM-dependent, and demonstrate that dCK activation is likely not carried out by functionally related serine/threonine kinases.

### Ser^74^ is critical for dCK activation by IR

**Figure 2:**
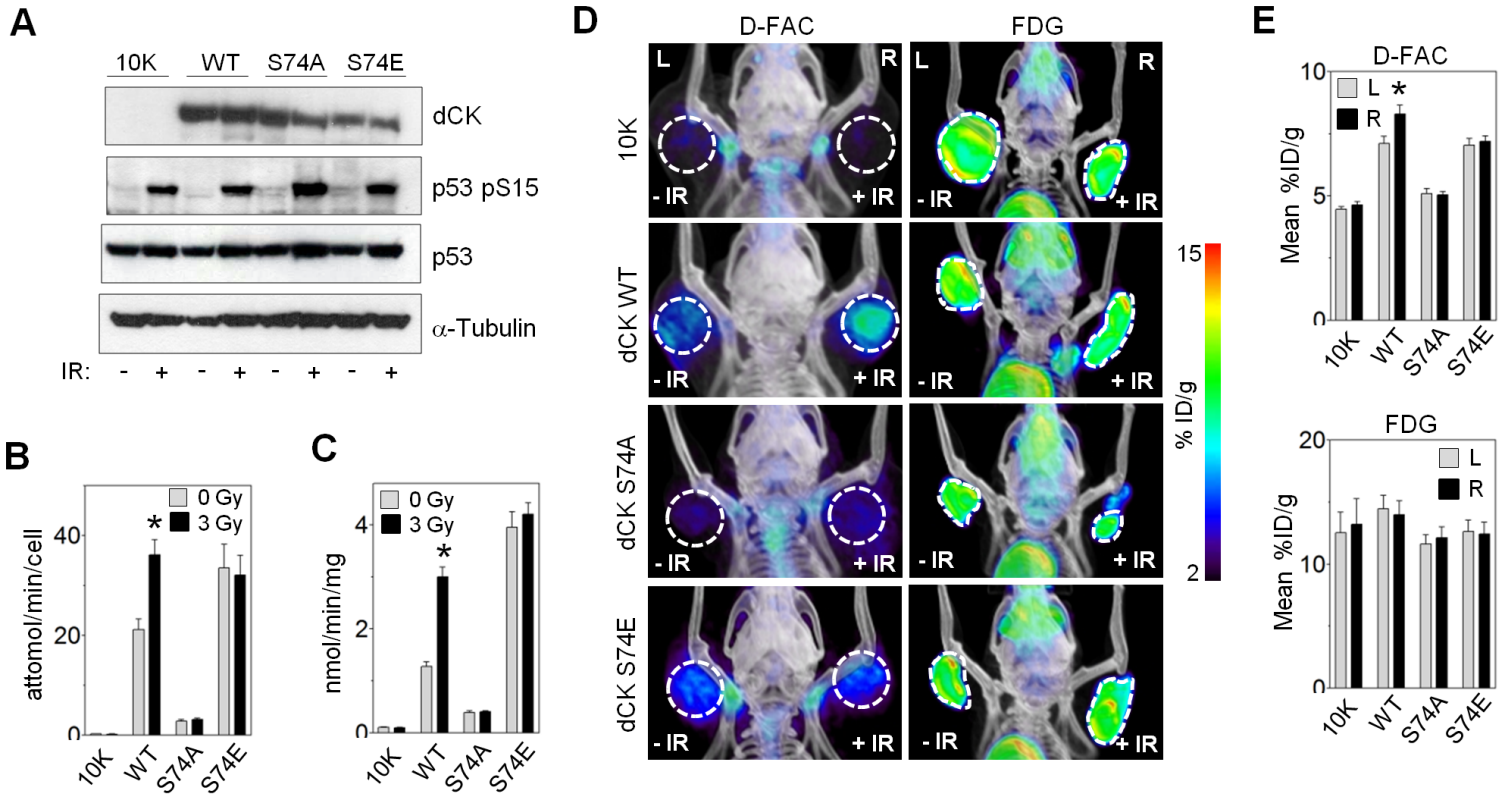
PET imaging of metabolic regulation during DNA damage response. (A) Western blot of 10K ± dCK (WT, S74A, S74E) before or 2 hours after exposure to 3 Gy. (B) *In vitro* cell uptake assay of [^3^H]-dC after 3 Gy exposure of L1210 Gy exposure of L1210-10K ± dCK WT (*, P = 0.0004; N = 14) or dCK Ser^74^ mutants. (C) *In vitro* dCK kinase assay using [^3^H]-dC and cell lysates from10K ± dCK WT (*, P<0.0001; N = 9), dCK S74A or dCK S74E, 2 hours after 3 Gy irradiation. (D) [^18^F]-FAC and [^18^F]-FDG microPET/CT scans of NOD-SCID mice with bilateral 10K ± dCK(WT, S74A, S74E) tumors after 3 Gy irradiation of right tumor. (E) Averaged ROI values of [^18^F]-FAC (top panel) or [^18^F]-FDG (bottom panel) uptake in irradiated (R, right) and untreated (L, left) tumors (*, P = 0.0267; N = 4 mice per group mice per group).

Ser^74^ of dCK is phosphorylated in response to IR [31] (Figure 1). Mutation of Ser^74^ was previously shown to dramatically affect the baseline activity of dCK [19]. The serine to alanine (S74A) mutation prevents Ser^74^ phosphorylation and results in reduced dCK activity [19]. In contrast, the serine to glutamic acid (S74E) mutation mimics phosphorylation and results in enhanced catalytic activity [19]. We measured the effect of these positive and negative mutations at Ser^74^ on the activity of dCK after IR. We first tested whether S74A substitution would lead to a loss of ATM-dependent activation of dCK after IR. The mutated kinase was expressed in L1210-10K cells through retroviral transduction followed by FACS sorting of YFP^+^ cells with fluorescence intensity matched to that of the 10K+dCK WT cells. Similar expression levels of dCK WT and Ser^74^ mutants were confirmed by Western blot (Figure 2A). Compared to un-irradiated cells containing WT dCK, and in agreement with previous studies, expression of S74A-dCK dramatically decreased the rate of ^3^H-dC uptake and demonstrated a 3-fold reduction in enzyme activity in cell extracts (Figure 2B and C). Importantly, S74A is not a kinase-dead mutation. dCK activity measured in 10K+S74A-dCK cell extracts is significantly greater than that in 10K extracts. This mutation also did not affect the level of expression of the enzyme (Figure 2A). Moreover, previous enzyme kinetic analysis has shown that the *k*_cat_ for dC phosphorylation by the purified S74A-dCK is the same as that for the WT dCK [21]. It is possible that unlike the phosphorylated Ser^74^ residue, the S74A mutation favors a closed dCK state, preventing substrate binding and increasing *K*_M_ for dC. Two hours after 3 Gy IR, ^3^H-dC uptake and S74A-dCK activity remained unchanged in marked contrast to the WT-dCK (Figure 2B and C).

We next tested the effect of S74E substitution on the IR-induced dCK activation. We found that this mutation tripled dCK activity compared to un-irradiated WT (Figure 2C). Un-irradiated S74E mutants also exhibited an increased rate of ^3^H-dC uptake compared to the WT dCK cells (Figure 2B). IR did not affect the ^3^H-dC uptake or the kinase activity of the S74E mutant (Figure 2B and C). Based on our results analyzing Ser^74^ mutations with positive and negative effects on the baseline dCK activity, we conclude that dCK is activated primarily by ATM-dependent phosphorylation of Ser^74^ in response to IR-induced DNA damage.

### Quantitative PET imaging of metabolic response to DNA damage

Our results indicate that ATM enhances dCK activity through post-translational modification in response to IR-induced DNA damage. Measuring dCK activity after IR *in vivo* may provide information about cellular DNA damage response. PET probes specific for dCK [34] allow non-invasive prediction of tumor response to gemcitabine, a prodrug substrate of dCK [41]. Measuring the DNA damage response non-invasively via dCK activation could be a useful clinical tool for stratifying patients into responders versus non-responders during treatment with IR. This imaging strategy could also provide a useful biomarker for assessing the extent of irradiation of the target field, or predict a potential synergism of nucleoside analogs with radiotherapy [54]. We tested whether IR-induced ATM-dependent activation of dN salvage would result in accumulation of the dCK-specific PET probe, [^18^F]-FAC, in irradiated tumors. L1210-10K tumors expressing WT dCK or Ser^74^ mutants were grown bilaterally on the front legs of NSG mice, and the right front leg was selectively irradiated with 3 Gy followed by tail-vein probe injection one hour later. The presence of WT dCK caused the irradiated tumor to retain significantly more [^18^F]-FAC compared to un-irradiated tumor (Figure 2D and E, top panel; Figure S2A). Taking the [^18^F]-FAC accumulation in 10K tumor as a background signal, IR induced a forty percent increase in [^18^F]-FAC accumulation in tumors expressing WT dCK (Figure 2E, top panel). Neither the 10K tumors nor Ser^74^ mutants exhibited increased probe uptake after IR (Figure 2D and E, top panel).

We repeated this experiment with a different set of animals, which were imaged with [^18^F]-FDG. PET imaging with [^18^F]-FDG measures glycolysis and tumor viability, and is routinely performed clinically to diagnose and stage malignancy, and measure response to therapy [55]. Accumulation of [^18^F]-FDG by 10K tumors with WT or mutated dCK remained unchanged acutely after IR (Figure 2D and E, bottom panel; Figure S2B). Our results demonstrate that a new class of PET probes specific for dCK may be used to visualize and quantify the immediate metabolic response to genotoxic stress induced by IR.

### Catalytic activity of dCK for deoxycytidine is selectively increased after IR

**Figure 3:**
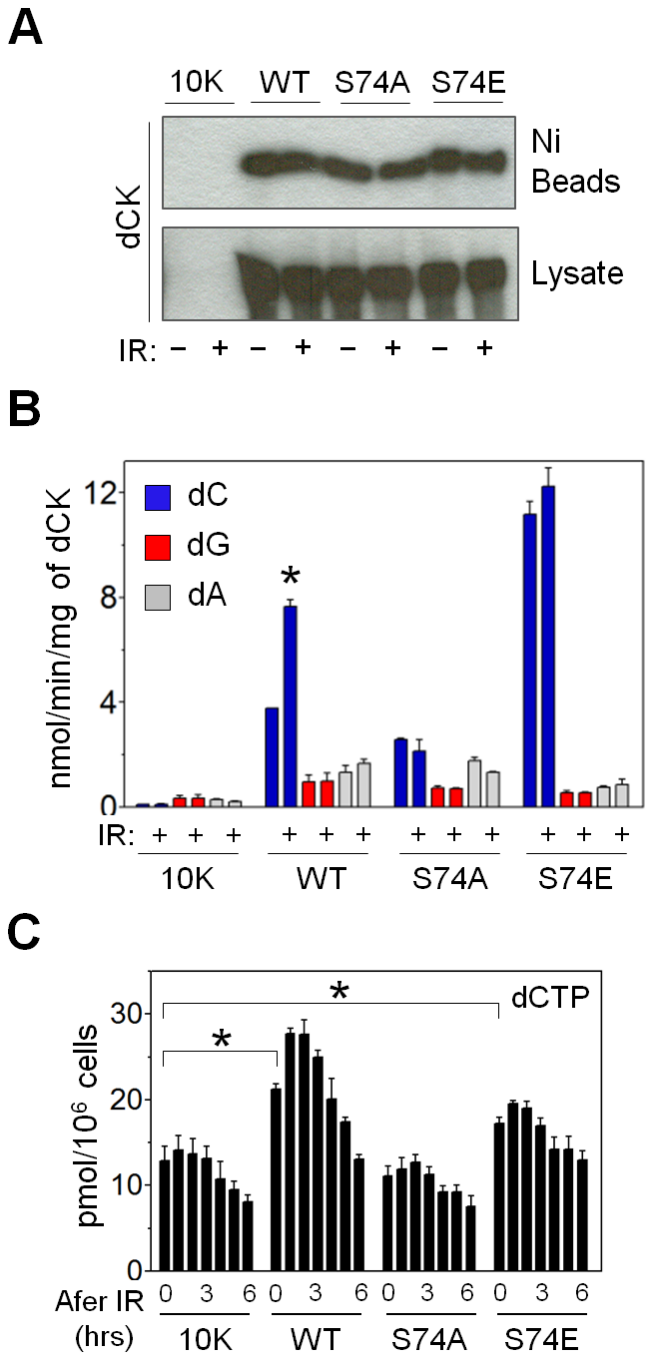
Changes in dCK substrate specificity and dCTP pool after IR. (A) Western blot of purified 6-His-tagged dCK (WT or Ser^74^ mutants) from 10K cells, and corresponding total cell lysates. (B) *In vitro* kinase assay on purified 6-His-tagged dCK (WT, S74A, S74E) using either [^3^H]-dC, [^3^H]-dG or [^3^H]-dA as a substrate before or after 3 Gy exposure (*, P = 0.0025; N = 2). (C) Intracellular dCTP pools before and hourly after 3 Gy exposure of 10K ± dCK (WT, S74A, S74E) cells (*, 10K vs. WT P = 0.0006; 10K vs. S74E P = 0.032; N = 6 for each time point).

Deoxycytidine kinase is able to phosphorylate pyrimidine (dC) as well as purines (dA, dG) [56]. Previous studies demonstrated that phosphomimetic S74E mutation increases catalytic activity of dCK for dC, but not for dA and dG [21], [22]. We hypothesized that ATM-dependent phosphorylation of dCK at Ser^74^ following IR serves to preferentially increase the production of dCMP over dGMP and dAMP. Because dG and dA are not exclusively phosphorylated by dCK, we generated histidine-tagged constructs of WT dCK and Ser^74^ mutants. Catalytic activities of affinity-purified His-tagged WT and mutated kinases for each dCK substrate were compared (Figure 3A). The rate of dC phosphorylation by the purified WT dCK doubled after IR, while the rates of dA and dG phosphorylation remained unchanged (Figure 3B). Confirming previous results, alanine substitution of Ser^74^ decreased dCK activity for dC, but had little effect on dA and dG phosphorylation. S74E-dCK tripled the rate of dC phosphorylation, while decreasing dG and dA phosphorylation rates by one half (Figure 3B). Both mutants exhibited no enzyme activity change for dG and dA after IR (Figure 3B). We conclude that IR-induced ATM-dependent phosphorylation of dCK at Ser^74^ increases the flux of deoxycytidine through the salvage pathway.

### Activated dCK increases dCTP production and the rate of DNA double-strand break repair

Intracellular pools of dNTPs must be tightly regulated to avoid genomic instability, while maintaining adequate supply for DNA replication and repair. Having determined that IR-induced dCK regulation by ATM enhances phosphorylation of dC, we measured the dNTP pools before and after radiation exposure. The concentration of dCTP at baseline was significantly higher when WT dCK or S74E dCK were expressed in L1210-10K cells compared to 10K cells (Figure 3C). Following IR the concentration of dCTP sharply rose in cells with WT dCK, reaching the maximum by two hours, and then falling below initial value six hours after irradiation. Neither the 10K cells nor the Ser^74^ dCK mutants exhibited a similar surge in dCTP. S74A substitution did not affect dCTP pools at baseline or after radiation compared to 10K cells (Figure 3C). An S74E substitution did not elevate dCTP concentrations as much as the WT-dCK (Figure 3C). Perhaps this reflects a different steady state of intracellular dNTP pools in cells expressing constitutively active dCK. We conclude that at least one function of ATM regulation of dCK may be to provide a source of dCTP for DNA repair.

**Figure 4:**
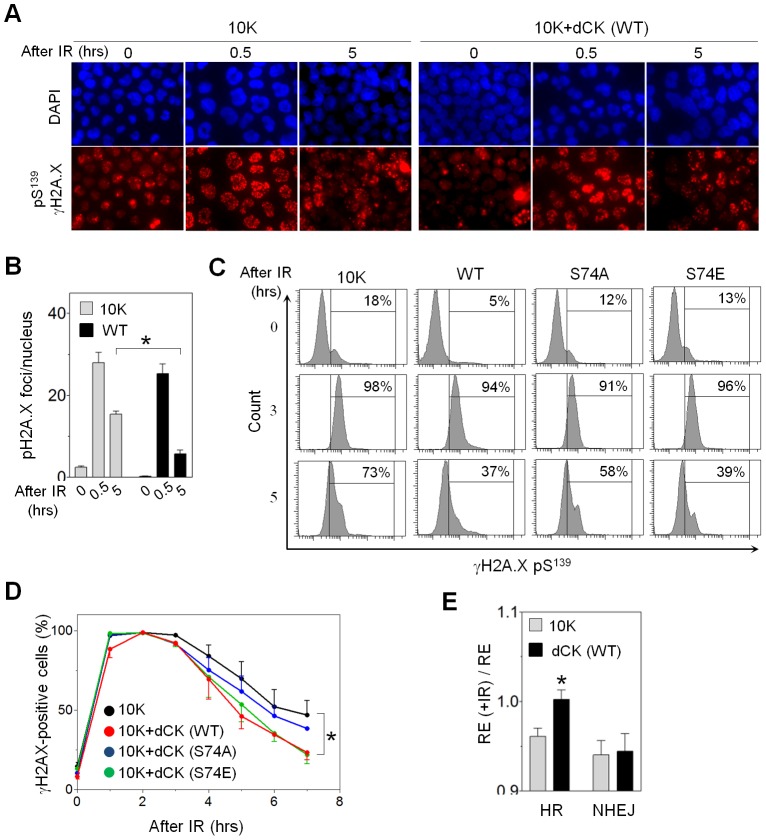
dCK influences the rate of IR-induced DNA repair. (A) Fluorescent images of DAPI and anti-pS^139^ γH2A.X stained 10K ± dCK (WT) cells before or 0.5 and 5 hours after exposure to 3 Gy. (B) Number of γH2A.X foci per cell nucleus (*, P<0.0001; N = 10) calculated from (A). (C) FACS analysis of pS^139^ γH2A.X positive 10K cells (indicated as % of total cell number) with or without dCK (WT, S74A, S74E) after 3 Gy irradiation. (D) Percentage of γH2A.X positive cells remaining over time after 3 Gy exposure (*, P = 0.01; N = 3 per time point per time point), obtained as shown in (C). (E) Recombination efficiency (RE) of digested plasmid via homologous recombination (HR) and non-homologous end joining (NHEJ) 24 hours after nucleofection of 3 Gy irradiated 10K ± dCK (WT) cells divided by the RE of the untreated cells (*, P = 0.0078, N = 10).

Most types of DNA repair require dNTPs, which may be salvaged from intracellular or exogenous sources, or synthesized *de novo*. It is possible that disabling dN salvage may slow DNA repair by requiring cells to rely solely on the *de novo* dNTP synthesis. Having determined that phosphorylation of dCK enhances dCTP production and pool size, we determined dCK-dependent kinetics of DNA DSB repair by measuring H2A.X phosphorylated at Ser^139^, a marker of DSB-induced DNA damage response. Resolution of pSer^139^ H2A.X foci (γH2A.X) corresponds to successful repair of DNA DSBs [23]. Before radiation, 10K cells contained 8 fold more γH2A.X foci then 10K+dCK cells (Figure 4A). 10K and 10K+dCK cells showed similar numbers of γH2A.X foci per nucleus 30 min after IR. At 5 hours post irradiation there were significantly fewer positive foci per nucleus in the cells expressing dCK (Figure 4A and B). We utilized flow cytometry to confirm these results and measure the effect of Ser^74^ mutation on DNA DSB repair rate. Again, the resolution of γH2A.X signal was significantly faster in the cells with WT dCK (Figure 4C and D). S74A substitution slowed DNA DSB repair rate, while S74E mutants were just as efficient at repairing DNA as the cells with WT dCK (Figure 4C and D).

Eukaryotic cells repair DNA DSB through either non-homologous end-joining (NHEJ) or homologous recombination (HR) [57]. Unlike NHEJ, HR requires extensive DNA synthesis, and is, therefore, dependent on the presence of sufficient dNTPs [58]. Blocking *de novo* dNTP synthesis via RNR inhibition suppresses HR [59]. Matsuoka, et al. observed that depletion of dCK in osteosarcoma U2OS cells by small interfering RNA increased baseline γH2A.X signal and compromised HR [31]. We tested the functionality of HR and NHEJ in 10K cells by utilizing a previously reported linearized plasmid transient transfection assay [42], [43]. The plasmid can be introduced into the cells immediately after IR, avoiding unpredictable IR-induced damage to the plasmid. Cells were irradiated one hour prior to plasmid transfection to induce DNA DSBs and DNA damage response, and recombination efficiency (RE) after IR was calculated and normalized to the recombination efficiency before IR utilizing flow cytometry data. Cells reconstituted with WT dCK exhibited a higher efficiency of plasmid recombination via HR compared to 10K cells (Figure 4E). Non-homologous end-joining, being relatively insensitive to dNTP levels, was not affected by the non-functional dN salvage (Figure 4E). We conclude that, consistent with previously published results [31], ATM-dependent phosphorylation of dCK increases the rate of DNA DSB repair by supplying dCTP for HR.

### Deoxycytidine salvage modulates radiosensitivity

**Figure 5:**
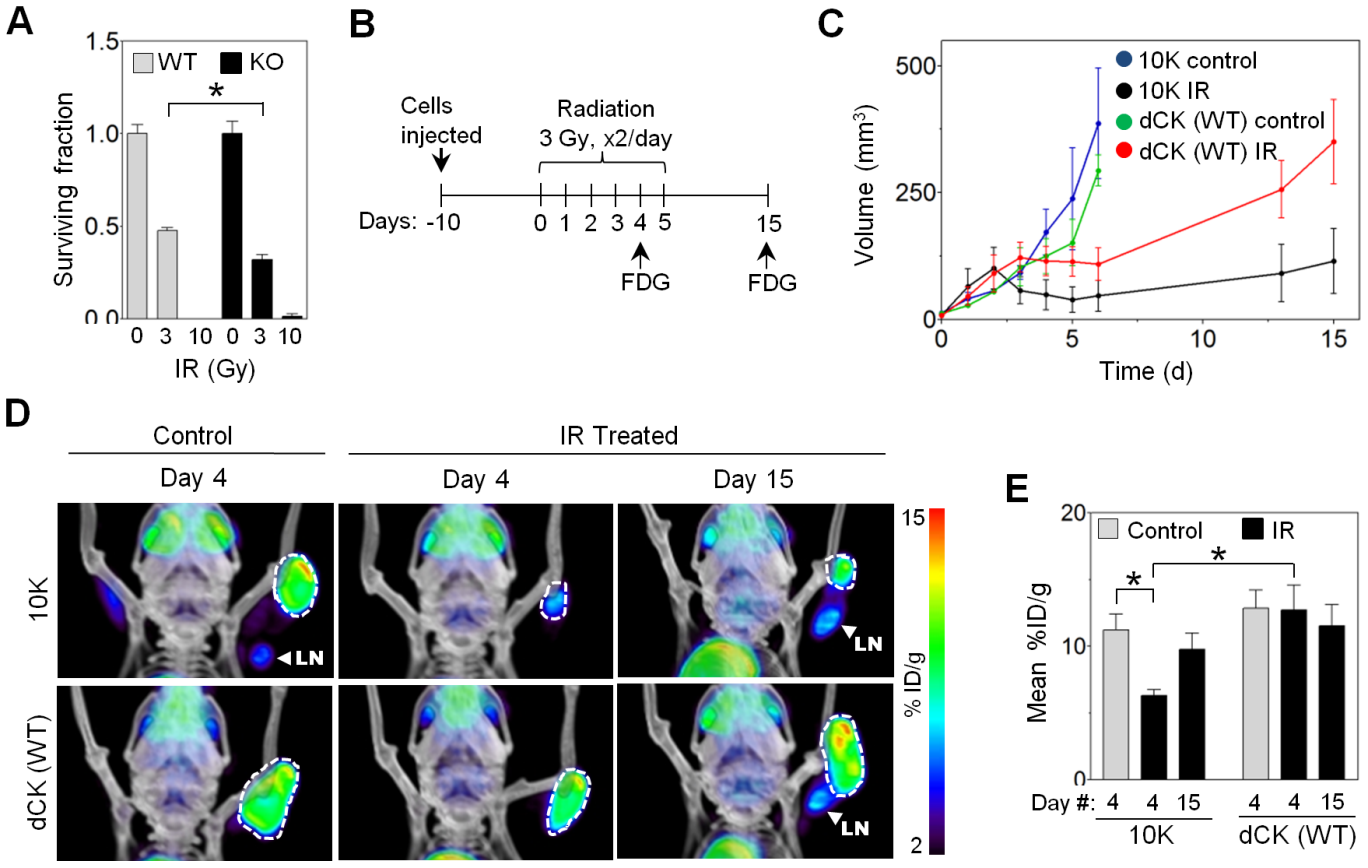
dCK absence sensitizes 10K cells and MEFs to IR. (A) Clonogenic survival assay of MEFs from dCK KO and dCK WT mice (*, P = 0.0383, N = 2). (B) Schematic of *in vivo vivo* IR treatment schedule (FDG refers to PET imaging). (C) Volume measurements of *in vivo vivo* untreated control and irradiated tumor model based on (L×W^2^)/2 formula (N = 4 mice per group). (D) [^18^F]-FDG microPET/CT scans of CB17 SCID mice treated as shown in (B) or untreated control. LN – lymph node. (E) Quantification of [^18^F]-FDG uptake in irradiated and untreated 10K ± dCK *in vivo vivo* tumor model (*, 10K ± IR at day 4: P = 0.0181; irradiated 10K vs. 10K+dCK at day 4: P = 0.0272; N = 4 mice per group).

Radiation therapy and a large number of chemotherapy drugs act by damaging cellular DNA. Interference with a cancer cell’s attempts to repair DNA further sensitizes it to therapy. The scope of strategies targeting DNA damage response in cancer is extremely broad [60]. Blockade of dNTP supply needed for replication and DNA repair is one such strategy. Inhibition of thymidylate synthase [61] and ribonucleotide reductase [62], [63], two critical enzymes of the *de novo* dNTP synthesis, has been utilized for tumor radiosensitization. The importance of dN salvage in IR-induced DNA repair makes this pathway a possible target for novel sensitizers to genotoxic therapy. Recently, Yang et al demonstrated that stable knock down of dCK in HeLa cells leads to a significant increase in IR radiosensitivity [33]. We also hypothesized that the absence of dCK will increase cancer radiosensitivity. Fibroblasts derived from dCK KO mice were significantly more radiosensitive to 3 Gy irradiation compared to MEFs with WT dCK (Figure 5A). We carried out an *in vivo* IR treatment study, implanting 10K cell grafts into front right legs of CB17 SCID mice. Noting that the rates of DSB repair were significantly different between 10K and 10K+dCK cells, we performed hyperfractionated radiation delivery utilized in clinical radiotherapy [64], delivering two 3 Gy doses daily. We hypothesized that 10K cells will be less efficient in repairing DNA between IR fractions, leading to accumulation of DNA damage and apoptosis. After a ten day engraftment, 36 Gy were delivered to the tumors in twelve fractions over six days (Figure 5B), followed by tumor re-growth. No differences in growth rates were observed between un-irradiated 10K and 10K+dCK tumors (Figure 5C). Tumors lacking dCK exhibited a greater reduction in volume in response to IR treatment compared to those expressing WT kinase (Figure 5C). In addition, PET imaging with [^18^F]-FDG on day 4 of IR treatment detected a significantly reduced viability and FDG uptake by tumors lacking dCK (Figure 5D and E). After completion of radiotherapy, the cancer relapsed as demonstrated by FDG-PET (Figure 5D and E), but 10K tumors remained smaller than those with WT dCK (Figure 5C and D). We conclude that the absence of dCK contributes to the enhanced radiosensitivity of L1210-10K murine leukemia cells and MEFs derived from dCK KO mice.

### Mutating Ser^74^ residue of dCK affects lymphocyte development

**Figure 6:**
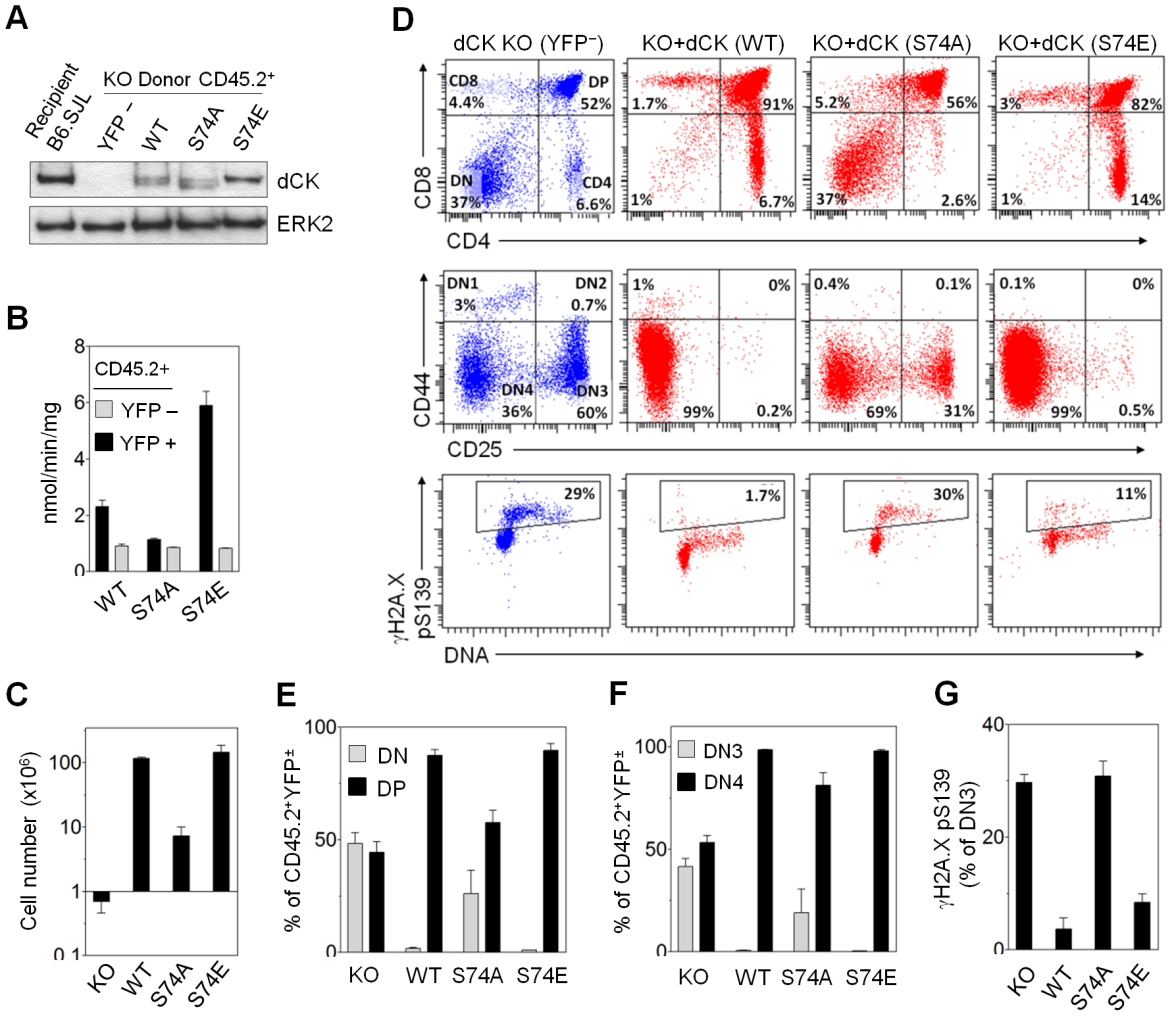
DCK Ser^74^ influences T cell development. (A) Western blot of CD45.2^+^ dCK KO cells (YFP^−^) carrying one of three dCK isoforms (WT, S74A, S74E; YFP^+^). Cells were isolated from recipient spleens and dCK expression level compared to endogenous dCK of CD45.1^+^ B6.SJL recipient. (B) *In vitro* dCK kinase assay using [^3^H]-dC and lysates of dCK KO donor CD45.2^+^ (dCK^+^YFP^+^ or dCK^−^YFP^−^) cells FACS purified from spleens of B6.SJL recipients (N = 3 mice per group). (C) Cellularity of B6.SJL thymi repopulated with dCK KO donor cells carrying different dCK isoforms (CD45.2^+^YFP^+^; N = 3). (D) FACS analysis of dCK KO ± dCK (WT, S74A, S74E) thymocytes. Bottom panels show pSer^139^ γH2A.X stained DN3 thymocytes. (E-F) Averaged percentages of single live donor CD45.2^+^ thymocytes (± dCK/YFP) at different stages of development (N = 3). (G) Averaged percentages of DN3 thymocytes positive for pSer^139^ γH2A.X (N = 3).

Thus far we described the response of dN salvage to the exogenous stress. Cells under endogenous, physiologic stress, resulting from metabolic production of reactive oxygen species (ROS) or DNA damage caused by rapid genomic replication [65], [66], may also activate dN salvage through dCK regulation. Thus, maintenance of adequate dNTP pools for DNA replication and/or repair during the development of rapidly dividing cells such as B and T cell precursors may be one important function of deoxyribonucleoside salvage. dCK KO mice accumulate DNA damage in lymphoid and erythroid cells undergoing replicative stress, as indicated by increased γH2A.X signal [37]. We hypothesized that the regulation of dCK through Ser^74^ phosphorylation observed *in vitro* may be important during lymphocyte development in mice. Bone marrow enriched for CD45.2^+^ hematopoietic stem cells (HSCs) was harvested from dCK KO mice [36], infected with retrovirus carrying YFP and either WT or Ser^74^-mutated dCK, and transplanted into lethally irradiated B6.SJL recipients (CD45.1^+^). Five to eight weeks after BMT, CD45.2^+^YFP^+^ lymphocytes from thymus and bone marrow were analyzed and compared to CD45.2^+^YFP^−^ dCK KO lymphocytes. We compared dCK expression in retrovirally transduced bone marrow to intrinsic dCK levels in WT bone marrow. Splenic donor CD45.2^+^YFP^+^ leukocytes expressed approximately one half the amount of WT or mutant dCK compared to CD45.1^+^ WT B6.SJL splenic leukocytes (Figure 6A). The activities of WT dCK and Ser^74^ mutants measured using total lysates from donor leukocytes were similar to those observed *in vitro* in 10K cell line (Figure 6B).

T cell development in the thymus proceeds through specific stages, which can be identified through distinct surface markers [67]. Immature thymocytes transition through CD4^−^CD8^−^ double negative (DN) stage (sub-categorized into DN1, DN2, DN3 and DN4) to CD4^+^CD8^+^ double positive (DP) stage to single positive (either CD4^+^ or CD8^+^) phenotype [67]. Thymi of dCK KO mice are severely hypocellular and contain a partial block within DN developmental stage, at DN3 to DN4 transition [36]. Upon successful completion of TCR β gene rearrangement and β-selection check point in DN3a, thymocytes undergo extensive proliferation in DN3b, DN4 and DP stages [68]. We analyzed thymic development of CD45.2^+^ dCK KO bone marrow reconstituted with WT or Ser^74^ mutant dCK and YFP marker. Re-expression of WT dCK in KO HSCs rescued the impaired thymic development as evidenced by normalized cellularity (Figure 6C), overall predominance of DP cells in the thymus, and DN stage consisting almost exclusively of DN4 cells (Figure 6D–F). Presence of S74E mutant similarly led to the reversal of abnormal dCK KO thymic development (Figure 6C–F). The development of S74A-dCK expressing thymocytes phenocopied dCK KO cells, but the impairment was less pronounced (Figure 6C–F). Cellularity of S74A thymi was an order of magnitude lower compared to the WT (Figure 6C). The partial block in DN3 to DN4 transition was not as severe with S74A-dCK as in KO mice (Figure 6D–F). Therefore, the activity of dCK, dictated by the Ser^74^ residue, influences T-cell development during bone marrow transplant reconstitution.

By staining DN3 thymocytes for pSer^139^ γH2A.X, we also measured whether altering dCK activity through Ser^74^ mutation affects the accumulation of DNA DSBs during T cell development. Accumulation of γH2A.X observed in DN3 thymocytes of KO and S74A-dCK animals was 8.5-fold higher compared to WT and 3.7 fold higher compared to S74E-dCK (Figure 6G), consistent with previous demonstration of enhanced DNA damage in dCK KO, but not WT, DN3 lymphocytes [37]. We conclude that hypofunctional, non-activatable S74A dCK leads to the accumulation of DNA double-strand breaks during thymocyte development.

**Figure 7:**
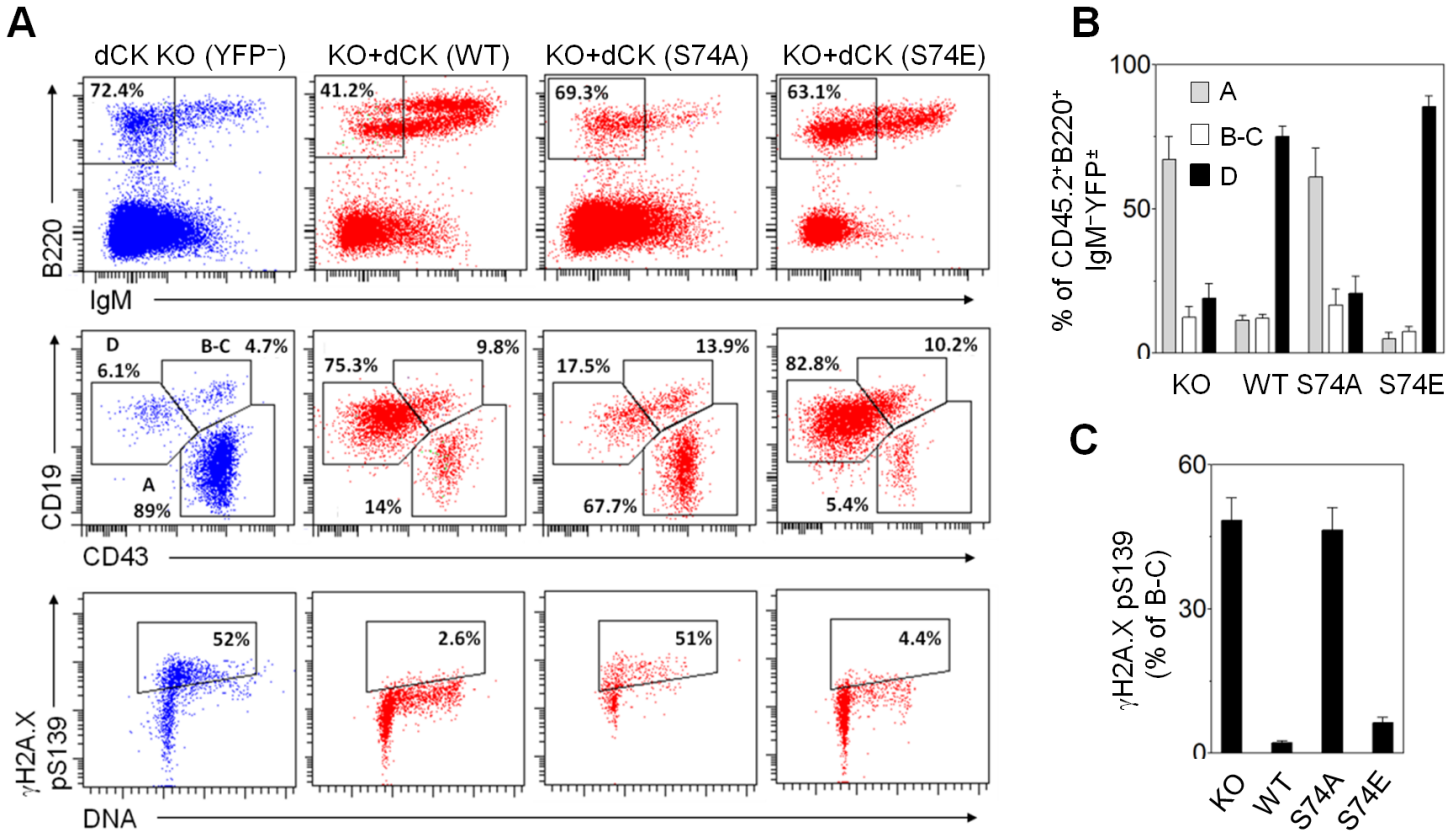
DCK Ser^74^ influences B cell development. (A) FACS analysis of CD45.2^+^ donor dCK KO ± YFP/dCK (WT, S74A, S74E) B cell development in the bone marrow of B6.SJL recipients. FACS plots are representative examples. Bottom panels show γH2A.X stained B–C Hardy fractions. (B) Averaged percentages of single live donor CD45.2^+^B220^+^IgM^−^ (± dCK/YFP) B cells at different stages of development (N = 3). (C) Averaged percentages of Hardy B–C fraction B cells positive for pSer^139^ γH2A.X (N = 3).

B cells at various stages of development may also be phenotyped based on the expression of specific surface markers [69], [70]. dCK KO mice have a partial block at the pro-B (CD43^+^CD19^+^) to pre-B cell (CD43^−^CD19^+^) transition [36]. At this stage, immature B cells are involved in immunoglobulin (Ig) gene rearrangement followed by clonal expansion [70]. Bone marrow reconstituted with the S74A-dCK mutant again phenocopied B-cell impairment in dCK KO mice (Figure 7A and B). Wild type dCK and the S74E mutant both rescued the dCK KO B-cell phenotype. Analysis of Hardy fractions [69] B–C (B220^+^IgM^−^CD19^+^CD43^+^), which include pro-B to pre-B transition, revealed significant accumulation of γH2A.X in the KO and S74A-dCK pro-B lymphocytes, but not in the pro-B cells with WT or S74E dCK (Figure 7A and C). Consistent with described DNA damage in developing dCK KO B cells [37], we demonstrate that dCK mutation at a single Ser^74^ residue is sufficient to impair B-cell development and cause accumulation of DNA DSBs in bone marrow reconstitution.

## Discussion

### The functional role of deoxycytidine kinase in DNA repair

Our results show that IR induces significant changes in deoxycytidine metabolism by activating dCK and increasing the salvage of deoxycytidine from the extracellular space in L1210-10K murine leukemia cell line. We show that ATM, but not ATR or DNA-PKcs, is critical for dCK activation by phosphorylating dCK at Ser^74^. The importance of ATM in dCK activation that we observe agrees with a recent study by Yang et al who also show that ATM phosphorylates dCK at Ser^74^ after IR [33]. The authors of the study conclude that the significance of this post-translational modification is to enable dCK to participate in an inhibitory protein complex with Cdk1 in the context of DNA damage, thereby establishing the G2M checkpoint. This result posits an interesting role for dCK that is independent of its function as a metabolic enzyme. Here, we do not address the role of dCK in protein complexes. It is possible that activated dCK has a dual role after IR to ensure effective DNA repair: (1) to increase production of nucleotide precursors for DNA repair machinery, and (2) to stop cell-cycle progression and allow time for DNA repair to take place. Further investigations using kinase-dead dCK mutants or specific inhibitors of dCK enzymatic activity are necessary to dissect the role of dCK in protein complexes from its metabolic function during DNA damage repair.

### Regulation of nucleotide precursor metabolism in response to DNA damage

ATM has been implicated in affecting various cytoplasmic processes in addition to its more established role in the nucleus [71]. One particular emerging role of ATM in the cytoplasm appears to involve the regulation of cellular metabolic pathways [71]. We observed that dCK localizes in the cytoplasm of CHOC6 and L1210 cell lines (Figure S3). After IR, dCK was not observed in the nucleus, and its enhanced activity was only evident in the cytoplasm (Figure S3). ATM was detected in the cytoplasm of CHOC6 cells only after IR, consistent with accumulating evidence of the extranuclear shuttling of ATM [71]. We believe, therefore, that our data suggests yet another example of ATM function outside of the nucleus.

Maintenance of adequate and balanced dNTP pools is an important task that cells execute to avoid genomic instability during DNA damage response. DSB repair through HR, in particular, requires the presence of sufficient dNTPs to synthesize long stretches of DNA after 5′- end resection [58]. To maintain dNTP pools under endogenous or exogenous stress, cells must co-regulate the *de novo* and salvage dNTP production. While the *de novo* dNTP synthesis undoubtedly plays an important role in DNA repair, the contribution of deoxyribonucleoside salvage to DNA repair has been less well explored [1]. However, increasing evidence of co-regulation and feedback between these two pathways is emerging. For example, RNR subunit p53R2 suppresses MEK-ERK activity [72], while inhibition of MAPK pathway activates dCK [13]. RNR is allosterically regulated by dTTP produced by the salvage pathway, shifting the specificity from pyrimidine to purine nucleotide reduction [73]. Finally, ATM regulates *de novo* dNTP synthesis through Ser^72^ phosphorylation of RNR p53R2 subunit [26], [27]. Mutation of that residue to alanine reduces basal and UV-induced dNTP levels [27]. While long-term dNTP balance may be achieved by increasing the expression of rate-limiting enzymes of dNTP synthesis, the majority of DNA DSB damage is repaired within the first 8 hours following the genotoxic insult. It is not surprising then that ATM regulates both *de novo* and salvage dNTP syntheses by post-translational modification of the rate limiting enzymes of these metabolic pathways, leading to a rapid increase of dNTP pools. Furthermore, each pathway may be responsible for supplying specific dNTPs for DNA repair. We show that the substrate specificity of phosphorylated dCK after IR shifts toward dC. Others demonstrated that the blockade of RNR by hydroxyurea causes selective depletion of purines (dATP and dGTP), but not pyrimidines [30]. ATM appears to coordinate the supply of dNTPs for DNA repair, and further work should aim at uncovering additional details of this regulatory mechanism and specific *de novo* and salvage pathway contributions.

Building on the previous evidence, we demonstrated that in L1210 murine leukemia cell line the rate limiting enzyme in dN salvage, dCK, is activated via phosphorylation at Ser^74^ by ATM in response to oxidative stress and IR-induced DNA damage. It is not clear whether oxidative stress can lead to dCK activation without inducing DNA damage. ATM is recruited to DNA double-strand breaks through association with Mre11-Rad50-Nbs1 (MRN) complex [74]. While MRN-dependent ATM autophosphorylation and monomerization has been one established mechanism of ATM activation, a separate mechanism involving direct oxidation and formation of an ATM dimer has been described [28]. Separating ROS production from DNA damage is difficult, and additional experiments are needed to further understand what activated form of ATM is responsible for regulating dN salvage pathway. We have observed that increased endogenous level of phosphorylated ATM at Ser^1981^ in ATR-Seckel cells compared to WT LCL, as also reported by others [75], does not result in dCK activation in the absence of IR (Figure 1F). This suggests that phos-Ser^1981^ modification of ATM is not sufficient for dCK activation. Furthermore, given the multitude of genotoxic agents which cause dCK activation, it remains unclear whether non-DSB forms of DNA damage such as single-strand DNA breaks lead to dN salvage activation, and whether this mechanism of activation is also ATM-dependent.

### Role of deoxyribonucleoside salvage pathway in endogenous stress response

The Ser^74^ residue of dCK is highly conserved across many species [19]. We hypothesized that ATM regulation of dCK is important in overcoming endogenous cellular stresses such as those resulting from oxidative species or DNA damage. Developing lymphocytes are particularly sensitive to the deletion of dCK [36]. Partial blocks in T and B lymphocyte development of dCK KO mice occur at the stages of VDJ and Ig gene recombination, respectively, and rapid proliferative expansion [67], [70], [76]. Reduced supply of dNTPs may slow programmed DNA repair and induce replication stress. However, gene rearrangement in developing lymphocytes occurs via non-homologous end joining pathway, which is relatively insensitive to dNTP levels [77]. Anabolic demands of major proliferative expansion result in increased metabolic activity, accumulation of reactive oxygen species and DNA damage [78]. It remains unclear whether dCK activation above baseline is needed to meet the demands of DNA replication and/or repair in the developing lymphocytes. ATM also plays an important role in VDJ recombination by activating cell-cycle checkpoints and directly stabilizing DNA DSB complexes [79]. Impairment of lymphocyte development in dCK KO mice is different from Atm^−/−^ mice. Defective processing of VDJ recombination in Atm^−/−^ thymocytes results in reduced production of mature CD4 and CD8 T cells and accumulation of double positive (DP) cells in the thymus [80]. Partially blocked transition from DN3 to DN4 developmental stage seen in dCK KO thymi [36] is not observed in Atm^−/−^ mice [80]. However, the regulation of dCK by ATM *in vitro* raises a possibility of similar mechanism occurring *in vivo*. The phenotype observed in T and B cell development with a mutation of a single Ser^74^ dCK residue suggests that regulation of dCK through Ser^74^ may play a role during hematopoiesis. Accumulation of γH2A.X foci in developing lymphocytes with KO dCK [37] and S74A-dCK mutants suggests that dCK activation based on post-translational modification may reduce endogenous genotoxic stress during clonal expansion. However, the S74A mutation compromises basal dCK activity. Mutating other residues critical for enzyme function which are not post-translationally modified should help determine whether reduced dCK activity exclusively accounts for the observed phenotype. Absence of a partial block at the double negative stage in Atm^−/−^ thymocytes may be due to a functional redundancy in dN regulation. For example, other members of PIKK family, ATR and DNA-PKcs may regulate dN salvage in response to single-strand DNA present at stalled replication forks or generated by processed DSBs.

### Exploiting the deoxyribonucleoside salvage pathway for cancer diagnosis and treatment

A tumor’s response to genotoxic stress is an important clinical diagnostic and prognostic factor. Efficient DNA damage repair in cancer cells may indicate a resistance to chemo- or radiation therapy. For this reason, clinical tools that can measure tumor responses to DNA damaging agents are necessary to give insight into therapeutic efficacy. Non-invasive quantitative imaging of the DNA damage response would be useful for stratification of patients and making necessary adjustments to the therapy. Using a dCK specific PET probe, we visualized and quantified IR-induced ATM-dependent activation of the dN salvage pathway, manifested as an increased probe accumulation in the tumor 1 hour after tumor irradiation. This is a unique demonstration of a clinically-relevant quantitative imaging read-out of the acute tumor metabolic response to genotoxic therapy. Higher PET signal after localized irradiation indicates enhanced dCK activation and a potential increase in DNA repair rate. These tumor responses also indicate that dN analog prodrugs like gemcitabine and clofarabine, which are phosphorylated into their active forms by dCK, could be used in synergy with radiotherapy [54]. Finally, this technology may delineate areas of a single tumor lesion with variable sensitivities to the therapy, as well as provide whole-body information in the cases of total body/bone marrow irradiation for cancer therapy and bone marrow transplantation. Future work will be aimed at determining specific tumor types which exhibit robust dCK activation and [^18^F]-FAC accumulation after IR.

In this study, we show that inhibition of deoxyribonucleoside salvage through the genetic deletion of dCK leads to enhanced radiosensitivity of a murine leukemia cell line and mouse embryonic fibroblasts. Yang et al showed enhanced radiosensitivity to IR in HeLa cells after stable dCK knockdown [33], suggesting that our result may be generalizable to other cell lines and types of human cancer expressing dCK. In principle, blockade of dCK by a small molecule inhibitor [81] may sensitize cancer to a wide range of genotoxic agents, including IR. Inhibitors of dCK Ser^74^ phosphorylation, in particular, may be useful in treating malignancy in conjunction with local radiotherapy. dCK is a promising target for cancer treatment and PET probes targeting dCK activity [35] will be useful in predicting and following clinical response to such therapy.

## Supporting Information

Figure S1 **IR-induced dCK activation is reduced in A-T cells.** (A) Western blot of CHOC6 (WT LCL) and A-T cell lines (AT224LA, AT255LA). (B) *In vitro* cell uptake assay of [^3^H]-dC by CHOC6, AT255LA and DK0064 cells before and 2 hours after exposure to 3 Gy (*, CHOC6: P = 0.034; DK0064: P = 0.023; N = 3). (C) Fold change in dCK activity of CHOC6, A-T cells (AT224LA, AT255LA) and DK0064 2 hours after 3 Gy (*, P<0.0001; N = 9).
(TIF)

Figure S2 **PET imaging of IR-induced ATM-dependent dCK activation.** (A) [^18^F]-FAC microPET/CT scans of four NOD-SCID mice with bilateral 10K+dCK (WT) tumors after 3 Gy irradiation of right tumor. Top two rows are coronal and transverse cross-sectional images, respectively. Bottom row: volume rendered images. (B) [^18^F]-FAC and [^18^F]-FDG microPET/CT scans of NOD-SCID mouse with bilateral 10 K tumors after 3 Gy irradiation of right tumor. Top row: coronal cross-section; bottom row: transverse cross-sectional images.
(TIF)

Figure S3 **dCK is localized and activated after IR in the cytoplasm.** (A) Western blot of nuclear (N) and cytoplasmic (C) fractions of CHOC6 (WT LCL) before and 2 hours after 3 Gy exposure. (B) *In vitro* dCK kinase assay using CHOC6 nuclear and cytoplasmic fraction lysates, [^3^H]-dC as substrate and performed 2 hours after exposure to 3 Gy (*, P = 0.0049, N = 3). (C) Western blot of nuclear (N) and cytoplasmic (C) fractions of L1210 cell line before and 2 hours after 3 Gy exposure. (D) *In vitro* dCK kinase assay using L1210 nuclear and cytoplasmic fraction lysates, [^3^H]-dC as substrate and performed 2 hours after exposure to 3 Gy (*, P = 0.0008, N = 3).
(TIF)
