# Supplementary figures and images for: Deoxycytidine Kinase Augments ATM-Mediated DNA Repair and Contributes to Radiation Resistance
Source: PLoS One. 2014 Aug 7;9(8):e104125. doi: 10.1371/journal.pone.0104125 (PMC4125169; doi:10.1371/journal.pone.0104125)

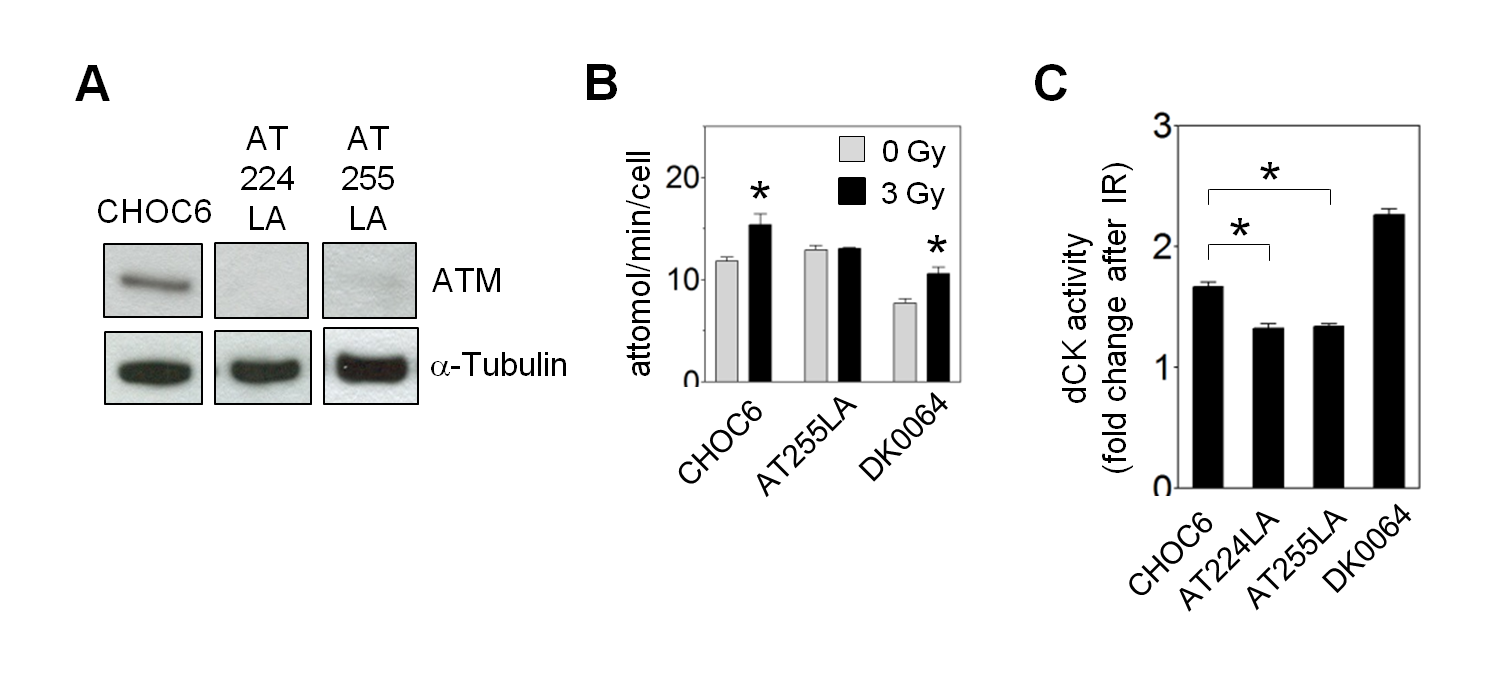

Supplement: Figure S1 — IR-induced dCK activation is reduced in A-T cells. (A) Western blot of CHOC6 (WT LCL) and A-T cell lines (AT224LA, AT255LA). (B) In vitro cell uptake assay of [3H]-dC by CHOC6, AT255LA and DK0064 cells before and 2 hours after exposure to 3 Gy (*, CHOC6: P = 0.034; DK0064: P = 0.023; N = 3). (C) Fold change in dCK activity of CHOC6, A-T cells (AT224LA, AT255LA) and DK0064 2 hours after 3 Gy (*, P<0.0001; N = 9). (TIF) [file pone.0104125.s001.tif]

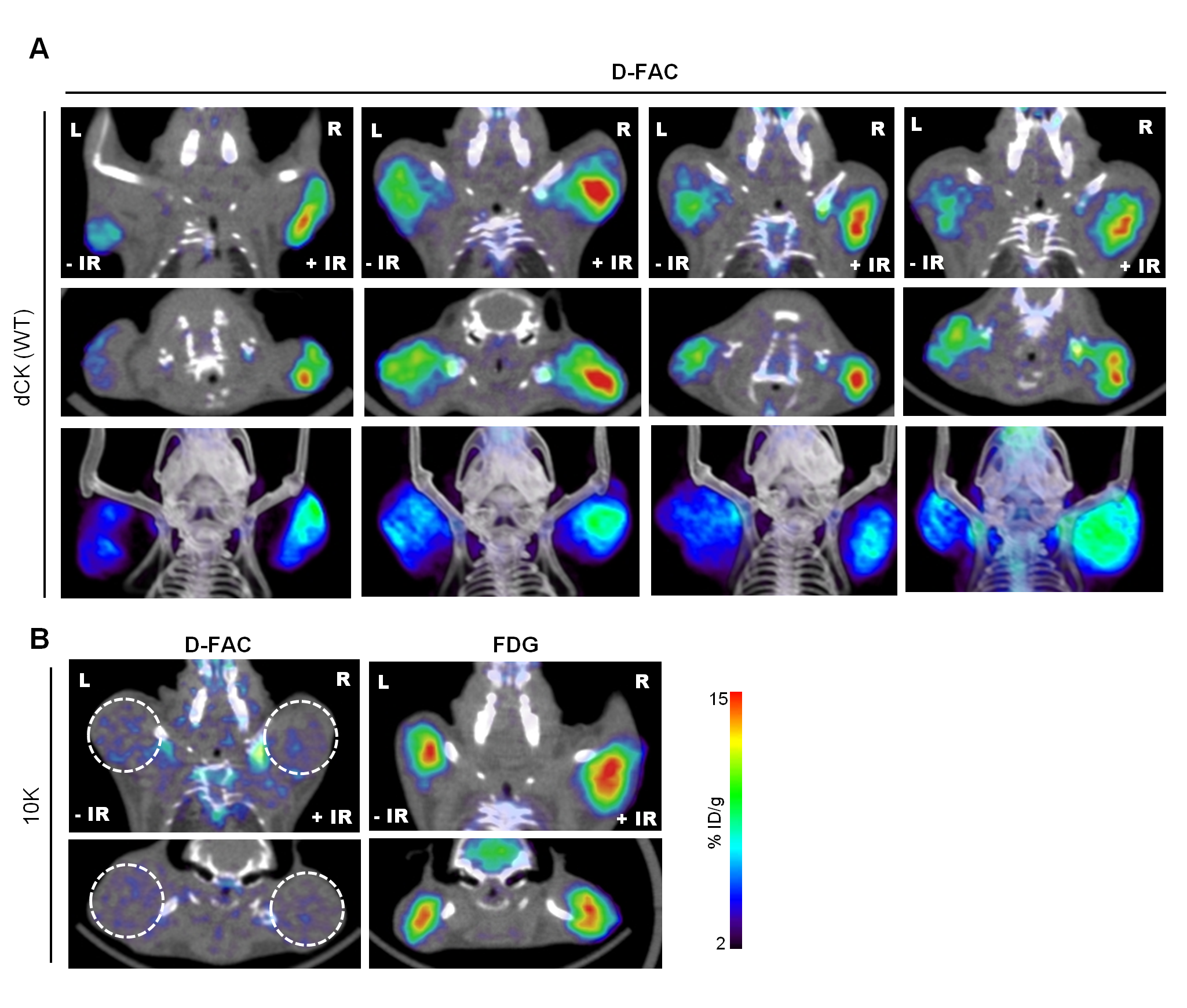

Supplement: Figure S2 — PET imaging of IR-induced ATM-dependent dCK activation. (A) [18F]-FAC microPET/CT scans of four NOD-SCID mice with bilateral 10K+dCK (WT) tumors after 3 Gy irradiation of right tumor. Top two rows are coronal and transverse cross-sectional images, respectively. Bottom row: volume rendered images. (B) [18F]-FAC and [18F]-FDG microPET/CT scans of NOD-SCID mouse with bilateral 10 K tumors after 3 Gy irradiation of right tumor. Top row: coronal cross-section; bottom row: transverse cross-sectional images. (TIF) [file pone.0104125.s002.tif]

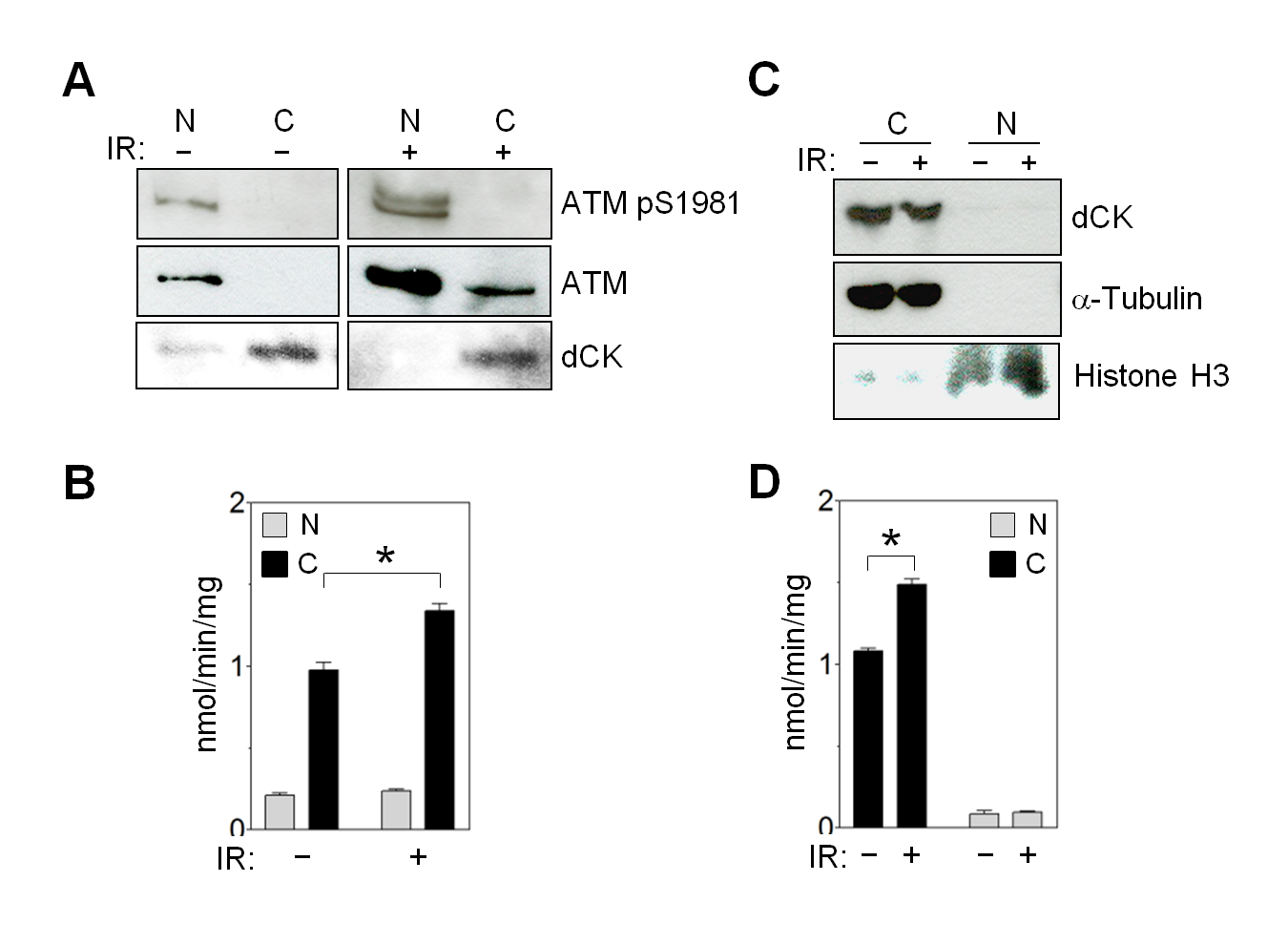

Supplement: Figure S3 — dCK is localized and activated after IR in the cytoplasm. (A) Western blot of nuclear (N) and cytoplasmic (C) fractions of CHOC6 (WT LCL) before and 2 hours after 3 Gy exposure. (B) In vitro dCK kinase assay using CHOC6 nuclear and cytoplasmic fraction lysates, [3H]-dC as substrate and performed 2 hours after exposure to 3 Gy (*, P = 0.0049, N = 3). (C) Western blot of nuclear (N) and cytoplasmic (C) fractions of L1210 cell line before and 2 hours after 3 Gy exposure. (D) In vitro dCK kinase assay using L1210 nuclear and cytoplasmic fraction lysates, [3H]-dC as substrate and performed 2 hours after exposure to 3 Gy (*, P = 0.0008, N = 3). (TIF) [file pone.0104125.s003.tif]
